# Risk of Pneumonitis and Pneumonia Associated With Immune Checkpoint Inhibitors for Solid Tumors: A Systematic Review and Meta-Analysis

**DOI:** 10.3389/fimmu.2019.00108

**Published:** 2019-02-04

**Authors:** Qiang Su, Emily C. Zhu, Jing-bo Wu, Teng Li, Yan-li Hou, Di-ya Wang, Zu-hua Gao

**Affiliations:** ^1^Department of Oncology, Beijing Friendship Hospital, Capital Medical University, Beijing, China; ^2^Desautels Faculty of Management, McGill University, Montreal, QC, Canada; ^3^The Fifth People's Hospital of Shanghai, Fudan University, Shanghai, China; ^4^Department of Pathology, Research Institute of McGill University Health Center, Montreal, QC, Canada; ^5^Department of Ophthalmology, Beijing Friendship Hospital, Capital Medical University, Beijing, China; ^6^Laboratory of Biorheology and Medical Ultrasonics, Hospital Research Center, University of Montreal, Montreal, QC, Canada

**Keywords:** pneumonitis, pneumonia, immune checkpoint inhibitors, meta-analysis, solid tumour

## Abstract

**Background:** We performed a systematic review and meta-analysis to evaluate the risk of pneumonitis and pneumonia associated with immune checkpoint inhibitors (ICIs) for solid tumors.

**Methods:** The following keywords were used in searching the Embase and PubMed database: pneumonitis, pneumonia, and immune checkpoint inhibitors. The data was analyzed by using the R software and Metafor package.

**Results:** Among 3,436 studies, 23 randomized clinical trials (RCTs) met our selection criteria which included data from **12,876** patients. Compared with chemotherapy, PD-1 inhibitors showed significant increase in grade 1-5 and grade 3-5 pneumonitis (RR, 5.17, 95% CI: 2.82–9.47, *p* < 0.001; RR, 4.14, 95% CI: 1.82–9.42, *p* < 0.001), but not in pneumonia. PD-L1 inhibitors showed significant increase in grade 1-5 pneumonitis and pneumonia (RR, 3.25, 95% CI: 1.61–6.57, *p* < 0.001; RR, 2.11, 95% CI: 1.20–3.70, *p* < 0.001). There was no significant difference in any grade pneumonitis and pneumonia in cytotoxic T lymphocyte-associated protein 4 (CTLA4) inhibitors subgroup. Programmed cell death protein 1 (PD-1) inhibitor (nivolumab and pembrolizumab) both showed significant increase in grade 1-5 pneumonitis, and pembrolizumab specially tended to increase grade 3-5 pneumonitis. (RR, 5.64 95% CI: 1.94–16.38, *p* < 0.001). Compared with PD-1 inhibitor (nivolumab) or CTLA-4 inhibitor (ipilimumab) monotherapy, PD-1 inhibitor, and CTLA-4 inhibitor (nivolumab plus ipilimumab) combination therapies showed significant increase in grade 1-5 and grade 3-5 pneumonitis (RR 3.47, 95%CI:1.76–6.83, *p* < 0.001; RR 3.48, 95%CI: 1.10–11.02, *p* < 0.001).

**Conclusions:** PD-1/PD-L1 inhibitors treatment could increase the risk of all-grade pneumonitis. CTLA4 inhibitor ipilimumab treatment alone could not increase the risk of pneumonitis but could augment the risk of pneumonitis in PD-1/PD-L1 inhibitor treated patients. There was no significant increase in the risk of pneumonia after either PD-1/PDL-1inhibitor or CTLA4 inhibitor treatment alone or in combination.

## Introduction

Immune-checkpoint targeted therapy using cytotoxic T lymphocyte-associated protein 4 (CTLA4), programmed cell death protein 1(PD-1) and programmed cell death ligand-1(PD-L1) inhibitors to overcome immune tolerance TOWARD cancer cells has been one of the major breakthrough in cancer therapy (1). Many controlled clinical trials had been carried out on immune checkpoint inhibitors(ICIs) therapy for tumor patients, and most of them resulted prolonged overall survival(OS), progression-free survival(PFS) or higher objective response rate(ORR). As of today, six ICIs have already been approved by FDA for clinical use, including one CTLA4 inhibitor (**ipilimumab**), two PD-1 inhibitors (**nivolumab and pembrolizumab**), and three PD-L1 inhibitors (**atezolizumab, durvalumab, and avelumab**).

The use of ICIs in cancer patients, however, is not without complications. Pneumonitis and pneumonia associated with ICIs are one of the most dangerous adverse events in cancer patients treated with ICIs. ICI-induced pneumonitis was defined as focal or diffuse immune-related inflammation of lung parenchymal cells after ICIs treatment. ICI-induced pneumonia was defined as the infection of lung caused by including Bacteria, fungus, or viruses after ICs treatment. Since the clinical application of ICIs, at least nine death cases related to ICI-induced pneumonitis (PD1/PD-L1: 7 cases; PD1 plus CTLA4: 2cases) and ten death cases of fatal pneumonia associated with ICIs (CTLA4: 7 cases; PD1/PD-L1: 3 cases) have been reported (Table 1). Awareness of the characteristics of pneumonitis and pneumonia associated with ICIs may aid in the appropriate utilization of ICIs in clinical practice, and appropriate monitoring of patients after ICIs treatment. This study was conducted to determine the relative risk of pneumonitis and pneumonia in patients with solid tumor treated with PD-1/PDL-1inhibitor or CTLA4 inhibitor alone or in combination.

**Table 1:** Difference of pneumonitis and pneumonia associated with Immune checkpoint inhibitors.

	**Pneumonitis**	**Pneumonia**
Cause	Immune checkpoint inhibitors	Infection including Bacteria, fungus, viruses
Symptoms	Shortness of breath, dry cough (occasionally, low-grade fever)	Coughing up sputum, shortness of breath, fever (or no sputum or fever)
Symptom duration	Weeks, months or even lifelong if it becomes chronic	A week to few months
Diagnosis	CT, PFT(pulmonary function test), lung biopsy	X-ray, blood and sputum culture, etc
Primary mechanism	Autoimmune(not clearly) and inflammation	pulmonary inflammatory response to mainly bacteria and bacterial products, etc
Treatment	Steroids, oxygen	Antibiotics, anti-viral, anti-fungal therapy, etc

## Results

### Selection of Studies

Using the search terminology, we initially identified 3,436 studies from our database search. Among those 3,436 studies, 23 RCTs met our strict inclusion criteria (Supplementary Figure 1). All the 23 included trials evaluated and compared the effectiveness of ICIs therapies with control treatments in solid tumors, representing data from a total of 12,876 patients (Table 2). Among the 23 studies, nine studies compared PD-1 inhibitors with chemotherapy (nivo: 5 studies, 1,128 patients; pem: 4 studies, 1,459 patients) (5–13), three studies compared PD-L1 inhibitors with control (atezolizumab: 2 studies, 751 patients; durvalumab:1 study, 475 patients) (2–4), four studies compared CTLA-4 inhibitors with control (tremelimumab: 2 studies, 705 patients; ipilimumab:2 studies, 864 patients) (14–17), three combined treatment of nivolumab and ipilimumab (522 patients) with nivolumab or ipilimumab (21–23), three compared combination treatment of ICIs and chemotherapy (3 studies, 694 patients) with chemotherapy (18–20), and one study compared pembrolizumab with ipilimumab (24). Night studies had data from malignant melanoma (MM) patients (6, 7, 13, 15, 16, 19, 21, 23, 24), nine from non-small cell lung cancer (NSCLC) patients (2–4, 8, 9, 11, 12, 18, 20), the other five from other cancers including small cell lung cancer (SCLC) (22), urothelial cancer (10), head-neck squamous cell carcinoma (HNSCC) (5), mesothelioma (14), and prostate cancer (17).

**Table 2:** Characteristics of the included randomized clinical trials.

**References**	**Study type**	**Histology**	**Endpiont**	**Treatment arms**	**Patients**	**Pneumonitis (grade 1-5, *n*)**	**Pneumonitis (grade 3-5, *n*)**	**Pneumonitis (grade 5, *n*)**	**Pneumonia (grade 1-5, *n*)**	**Pneumonia (grade 3-5, *n*)**	**Pneumonia (grade 5, *n*)**
Rittmeyer et al. (2)	RCT III PD-L1	NSCLC	OS	Atezolizumab 1,200 mg q3w	609	6	4	0	NA	20	0
				Docetaxel 75 mg/m2 q3w	578	1	1	0	NA	31	1
Fehrenbacher et al. (3)	RCT II PD-L1	NSCLC	OS	Atezolizumab 1,200 mg q3w	142	4	1	0	14	10	1
				Docetaxel 75 mg/m2 q3w	135	0	0	0	4	3	0
Antonia et al. (4)	RCT III	NSCLC	OS PFS	Durvalumab 10 mg/kg q2w	475	43	6	4	62	21	0
				Placebo	234	8	2	2	18	9	0
Ferris et al. (5)	RCT III PD-1	HNSCC	OS	Nivolumab 3mg/kg q2w	236	5	2	0	10	8	0
				Chemotherapy	111	1	0	0	1	1	1
Weber et al. (6)	RCT III PD-1	Melanoma	OS,ORR	Nivolumab 3 mg/kg q2w	268	5	1	0	NA	7	0
				Chemotherapy	102	0	0	0	NA	0	0
Robert et al. (7)	RCT III PD-1	Melanoma	OS	Nivolumab 3 mg/kg q2w	206	3	2	0	NA	1	0
				Dacarbazine (1,000 mg/m2) q3w	205	0	0	0	NA	0	0
Brahmer et al. (8)	RCT III PD-1	NSCLC	OS	Nivolumab 3 mg/kg q2w	131	2	1	0	0	0	0
				Docetaxel 75 mg/m2 q3w	129	0	0	0	1	1	0
Borghaei et al. (9)	RCT III PD-1	NSCLC	OS	Nivolumab 3 mg/kg q2w	287	8	3	0	0	0	0
				Docetaxel 75 mg/m2 q3w	268	1	1	0	5	5	0
Bellmunt et al. (10)	RCT III PD-1	Urothelial carcinoma	OS, PFS	Pembrolizumab 200 mg q3w	266	11	6	1	NA	9	0
				Chemotherapy	255	1	0	0	NA	8	0
Reck et al. (11)	RCT III PD-1	NSCLC	PFS	Pembrolizumab 200 mg q3w	154	9	4	0	NA	3	0
				Chemotherapy	150	1	1	0	NA	9	0
Herbst et al. (12)	RCT II/III PD-1	NSCLC	OS, PFS	Pembrolizumab 2 mg/kg q3w	339	14	6	1	5	3	1
				Pembrolizumab 10 mg/kg q3w	343	12	6	1	5	3	1
				Docetaxel 75 mg/m2 q3w	309	3	1	1	5	4	1
Ribas et al. (13)	RCT II PD-1	Melanoma	PFS	Pembrolizumab 2 mg/kg q3w	178	3	0	0	NA	3	0
				Pembrolizumab 10 mg/kg q3w	179	3	2	0	NA	3	0
				Chemotherapy	171	0	0	0	NA	6	0
Maio et al. (14)	RCT II CTLA4	Mesothelioma	OS	Tremelimumab 10mg/kg q4w -q3m	380	3	1	0	18	9	1
				Placebo	189	0	0	0	9	6	0
Ribas et al. (15)	RCT III CTLA4	Melanoma	OS	Tremelimumab 15 mg/kg q3m	325	NA	NA	NA	NA	NA	1
				Chemotherapy (temozoloide/dacarbazine)	319	NA	NA	NA	NA	NA	1
Eggermont et al. (16)	RCT III CTLA4	Melanoma	RFS	Ipilimumab 10 mg/kg q3w - q3m	471	NA	3	0	NA	3	0
				Placebo	474	NA	0	0	NA	1	0
Kwon et al. (17)	RCT III CTLA4	Prostate cancer	OS	Ipilimumab 10 mg/kg q3w	393	5	1	0	24	16	4
				Placebo	396	0	0	0	9	3	0
Govindan et al. (18)	RCT IV CTLA4+chemo vs. chemo	NSCLC	OS	Ipilimumab + chemotherapy	388	1	1	0	8	6	1
				Chemotherapy	361	2	2	0	3	2	0
Robert et al. (19)	RCT III CTLA4+chemo vs. chemo	Melanoma	OS	Ipilimumab + dacarbazine	247	NA	2	0	NA	5	0
				Dacarbazine	251	NA	0	0	NA	2	0
Langer et al. (20)	RCT II PD-1+chemo vs. chemo	NSCLC	ORR	Pembrolizumab + chemotherapy	59	3	1	0	1	1	0
				Chemotherapy	62	0	0	0	0	0	0
Wolchok et al. (21)	RCT III PD-1 + CTLA4 vs. CTLA4/PD-1	Melanoma	PFS,OS	Nivolumab + ipilimumab	313	22	3	0	NA	6	0
				Nivolumab	313	5	1	0	NA	0	0
				Ipilimumab	311	5	1	0	NA	2	0
Antonia et al. (22)	RCT I/ II PD-1+CTLA4 vs. PD-1	SCLC	ORR	Nivolumab 1 + ipilimumab 3	61	2	1	0	NA	NA	NA
				Nivolumab3 + ipilimumab 1	54	3	1	1	NA	NA	NA
				Nivolumab	98	3	1	0	NA	NA	NA
Hodi et al. (23)	RCT II PD-1 + CTLA4 vs. CTLA4	Melanoma	ORR	Nivolumab + ipilimumab	94	9	2	1	1	1	0
				Ipilimumab	46	0	0	0	0	0	0
Robert et al. (24)	RCT III PD-1 vs. CTLA4	Melanoma	PFS,OS	Pembrolizumab 10 mg/kg q2w	278	1	0	0	NA	3	0
				Pembrolizumab 10 mg/kg q3w	277	5	1	0	NA	1	0
				Ipilimumab 3 mg/kg q3w	256	1	1	0	NA	3	0

*RCT, randomized clinical trial; NSCLC, non-small cell lung cancer; PFS, progression-free survival; OS, overall survival; ORR, objective response rate, Chemo, chemotherapy, NA, not available*.

Cochrane risk of bias tool was used to measure the quality of the included studies and the results are shown in Supplementary Figure 2. All of the included studies have described the details in regard to blinding of outcome assessment, and random sequence generation. However, some of them had described incomplete outcome data and allocation concealment. Some studies failed to mention blinding of participants and personnel and selective reporting. Other indices of bias lacked specific description in all of the included clinical studies.

### Risk of Pneumonitis and Pneumonia in PD-1/PD-L1 and CTLA-4 Inhibitors

**Figure 1:**
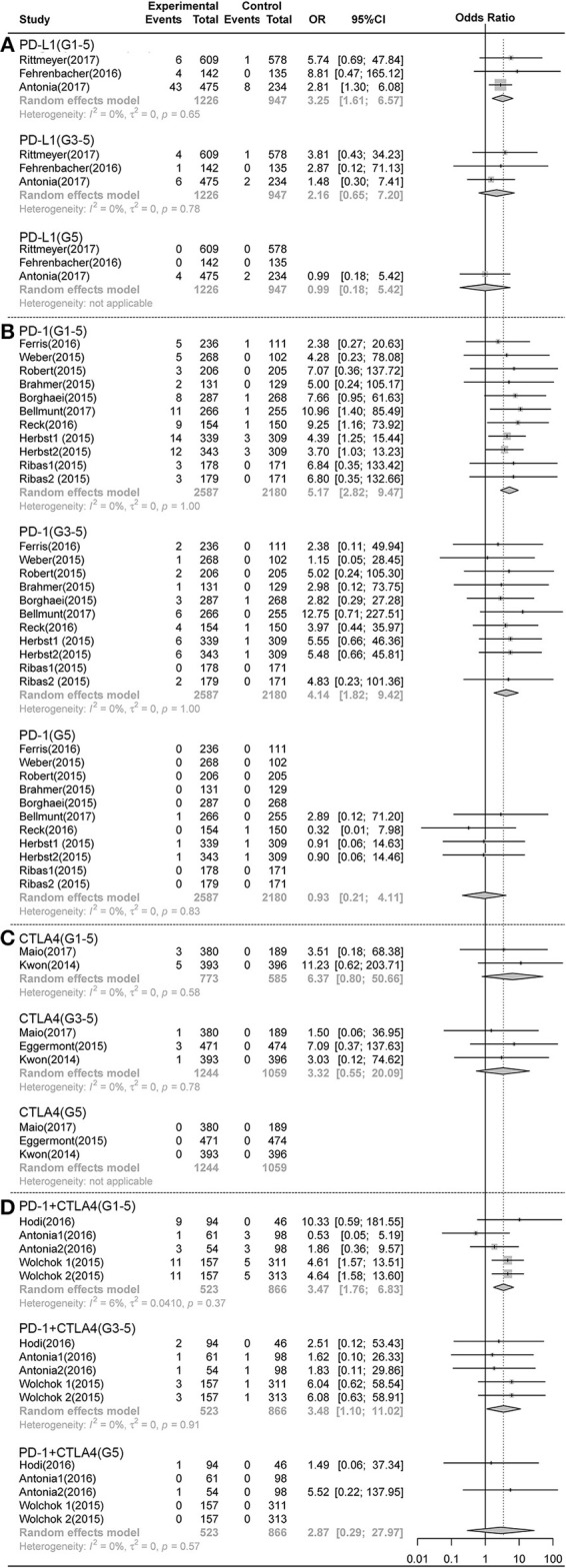
Forest plot analysis of pneumonitis comparing PD-1/PD-L1/CTLA4 with control therapies. **(A)** PD-L1 inhibitor V.S. chemotherapy/placebo; **(B)** PD-1 inhibitor V.S. chemotherapy; **(C)** CTLA4 inhibitor V.S. chemotherapy/placebo; **(D)** PD-1 combined CTLA4 V.S. ICI. **G1-5**, grade 1-5; **G3-5**, grade 3-5, **G5**, death.

**Figure 2:**
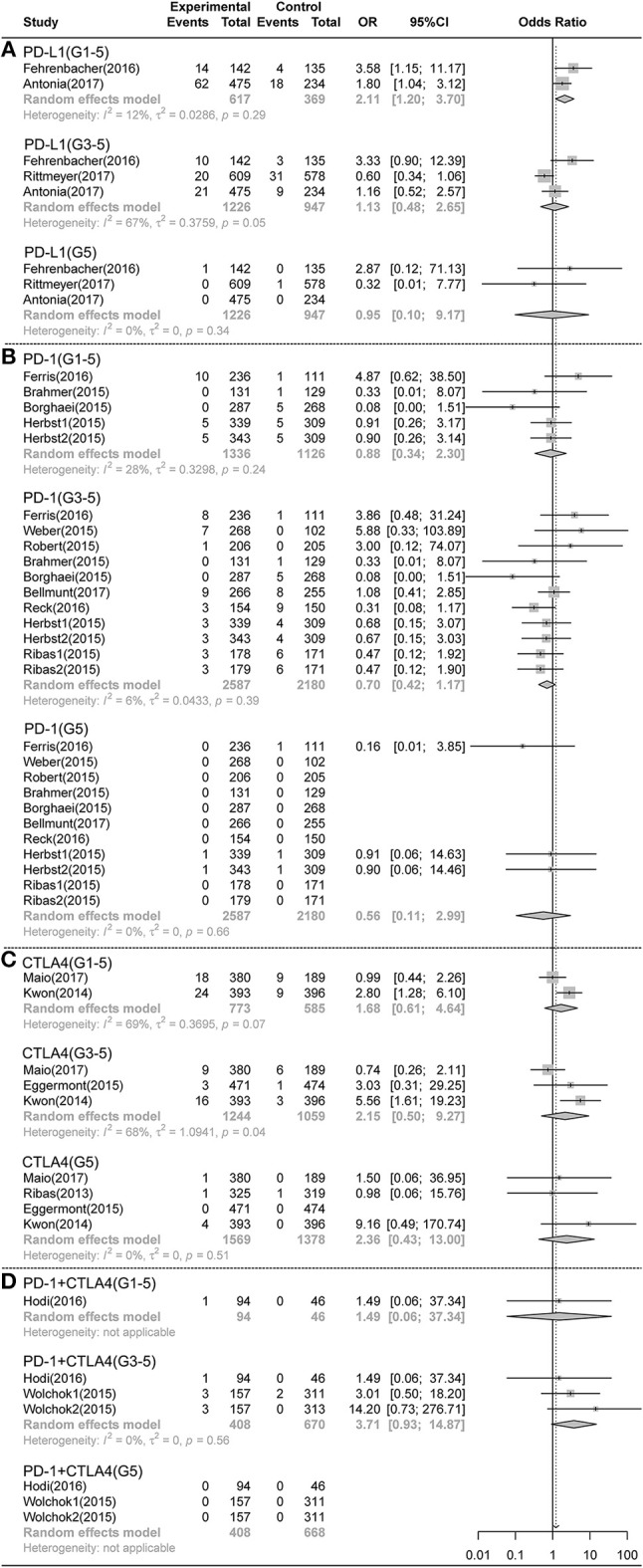
Forest plot analysis of pneumonia comparing PD-1/PD-L1/CTLA4 with control therapies. **(A)** PD-L1 inhibitor V.S. chemotherapy/placebo; **(B)** PD-1 inhibitor V.S. chemotherapy; **(C)** CTLA4 inhibitor V.S. chemotherapy/placebo; **(D)** PD-1 combined CTLA4 V.S. ICI. **G1-5**, grade 1-5; **G3-5**, grade 3-5, **G5**, death.

As shown in Figures 1, 2, compared with chemotherapy, PD-1 inhibitors showed significant increase in grade 1-5 and grade 3-5 **pneumonitis** (RR,5.17, 95% CI: 2.82-9.47, *p* < 0.001, RR,4.14, 95% CI:1.82-9.42, *p* < 0.001),but not in **pneumonia**. Compared with control, PD-L1 inhibitors showed significant increase in grade 1-5 **pneumonitis** and **pneumonia** (RR, 3.25, 95% CI: 1.61-6.57, *p* < 0.001, RR, 2.11, 95% CI: 1.20-3.70, *p* < 0.001). There was no significant difference in any grade **pneumonitis** and **pneumonia** in CTLA4 inhibitors subgroup.

Compared with PD-1 inhibitor (nivolumab) or CTLA-4 inhibitor (ipilimumab) monotherapy, PD-1 inhibitor and CTLA-4 inhibitor (nivolumab plus ipilimumab) combination therapies showed significant increase in grade 1-5 and grade 3-5 pneumonitis (RR 3.47, 95%CI:1.76-6.83, *p* < 0.001; RR 3.48, 95%CI:1.10-11.02, *p* < 0.001) (Figure 1), but not in pneumonia (Figure 2).

Compared with chemotherapy, there was no significant difference in the risk of any grade pneumonitis and pneumonia in ICIs plus chemotherapy combination therapies. When compared with CTLA-4 inhibitors, the risk of any grade pneumonitis induced by PD-1inhibitors seems higher, which however, was not statistically significant (Supplementary Figure 3).

### Risk of Pneumonitis and Pneumonia in Ipilimumab, Atezolizumab, Nivolumab, and Pembrolizumab

**Figure 3:**
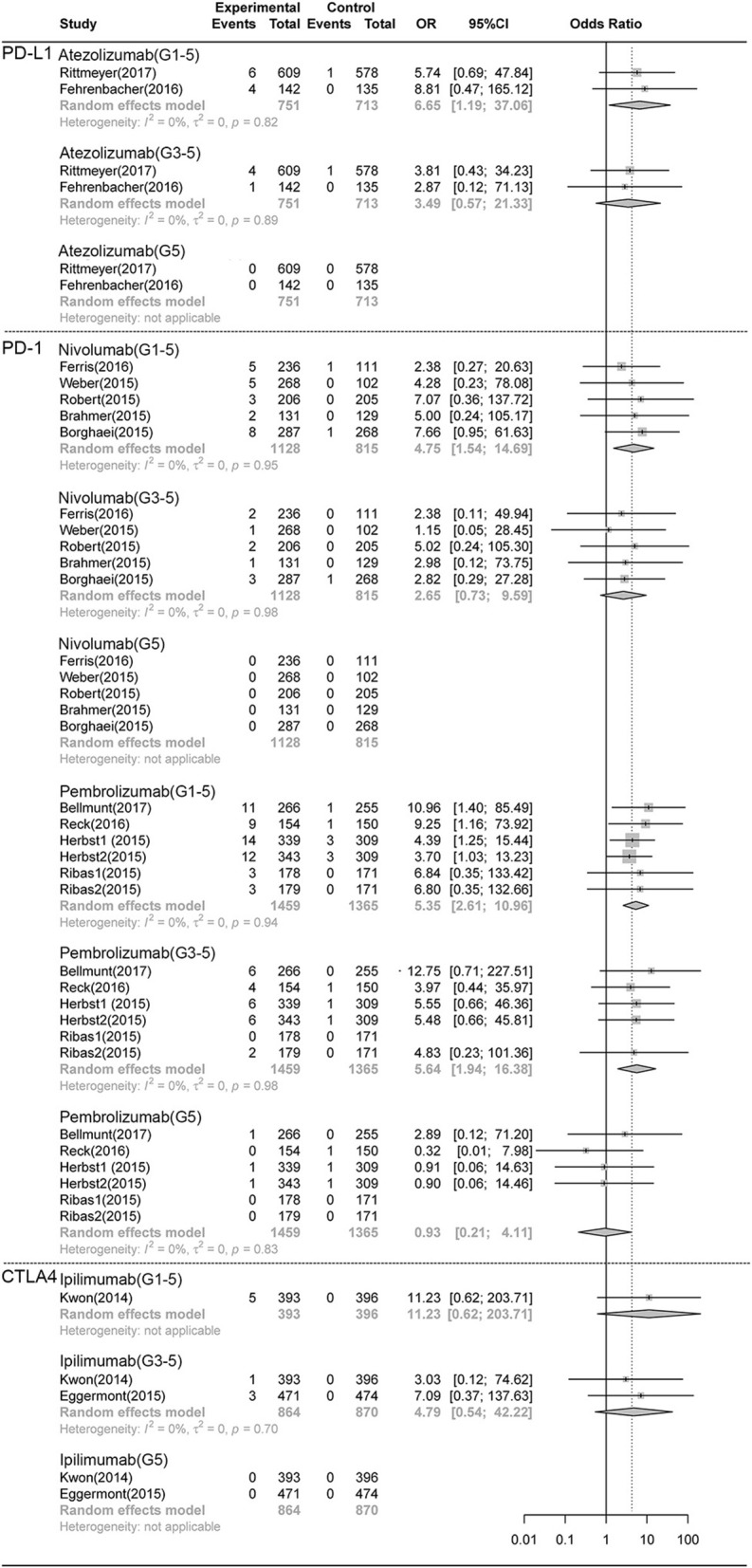
Forest plot analysis of pneumonitis comparing different ICIs with control therapies. **Atezolizumab**, atezolizumab V.S. chemotherapy; **Nivolumab**, nivolumab V.S. chemotherapy; **Pembrolizumab**, pembrolizumab V.S. chemotherapy; **G1-5**, grade 1-5; **G3-5**, grade 3-5, **G5**, death.

**Figure 4:**
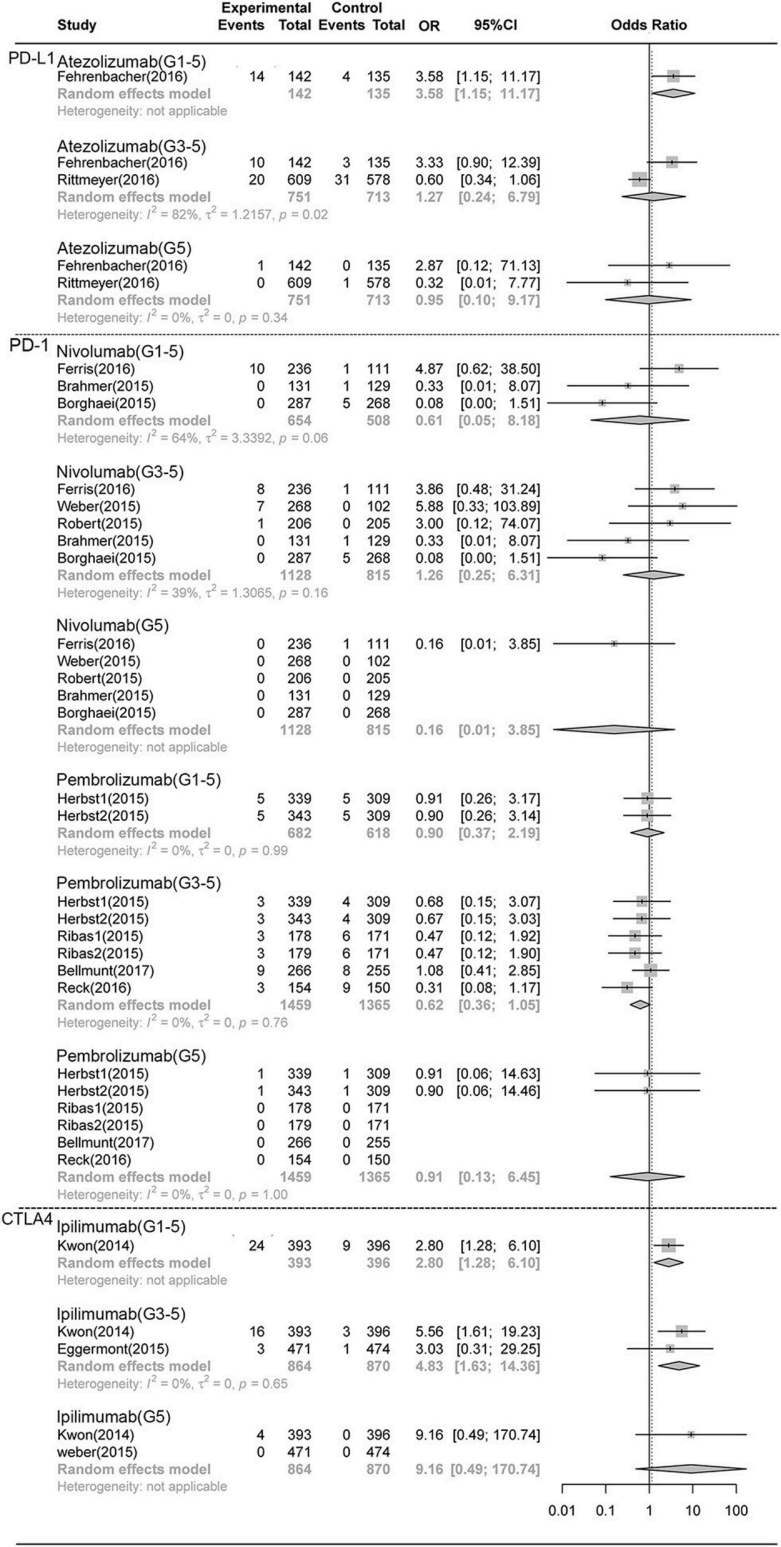
Forest plot analysis of pneumonia comparing different ICIs with control therapies**. Atezolizumab**, atezolizumab V.S. chemotherapy; **Nivolumab**, nivolumab V.S. chemotherapy; **Pembrolizumab**, pembrolizumab V.S. chemotherapy; **G1-5**, grade 1-5; **G3-5**, grade 3-5, **G5**, death.

As shown in Figures 3, 4, compared with chemotherapy, the PD-1 inhibitor nivolumab and pembrolizumab both showed significant increase in grade 1-5 **pneumonitis** (nivolumab: RR,4.75, 95% CI: 1.54-14.69, *p* < 0.001; pembrolizumab: RR,5.35, 95% CI:2.61-10.96, *p* < 0.001), but only pembrolizumab showed significant increase in grade 3-5 **pneumonitis**. (RR, 5.64 95% CI: 1.94-16.38, *p* < 0.001), while nivolumab did not show significant increase (RR 2.65, CI 0.73-9.59, P>0.05). There was no significant difference in grade 1-5 and grade 3-5 **pneumonia** in nivolumab or pembrolizumab subgroup. PDL-1 inhibitor Atezolizumab showed significant increase in grade 1-5 **pneumonitis** and **pneumonia** (RR,6.65, 95% CI: 1.19-37.06, *p* < 0.001; RR,5.35, 95% CI:2.61-10.96, *p* < 0.001, respectively).Compared with control, there was no significant difference in grade 1-5 or grade 3-5 **pneumonitis** in CTLA-4 inhibitor ipilimumab subgroup.

There was no significant difference in the risk of death (grade5 **pneumonitis** and **pneumonia**) between any ICI treatment group and control treatment group (Figures 1–4).

### Risk of Pneumonitis and Pneumonia in Different Tumoral Types With PD-1/PD-L1 and CTLA-4 Inhibitors

**Figure 5:**
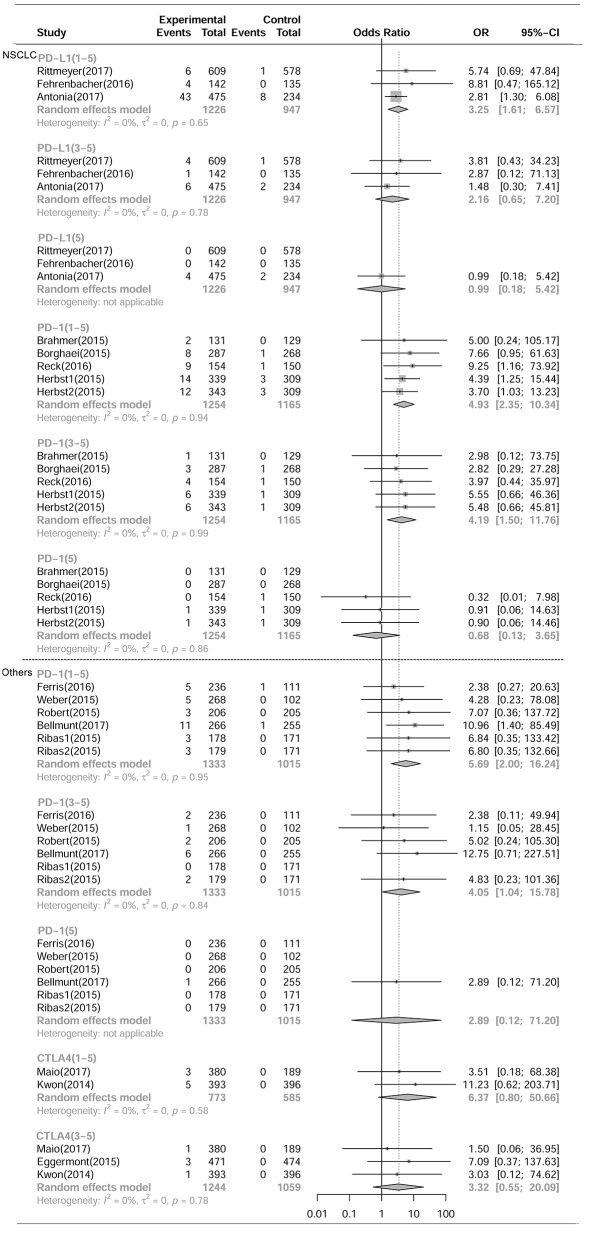
Forest plot analysis of pneumonitis of different ICIs in different tumoral types. **NSCLC**, non-small cell lung cancer; **Others**, including MM, HNSCC, Prostate cancer, Mesothelioma. PD-L1, PD-L1 inhibitor V.S. chemotherapy/placebo; PD-1, PD-1 inhibitor V.S. chemotherapy; CTLA4, CTLA4 inhibitor V.S. chemotherapy/placebo; **G1-5**, grade 1-5; **G3-5**, grade 3-5, **G5**, death.

**Figure 6:**
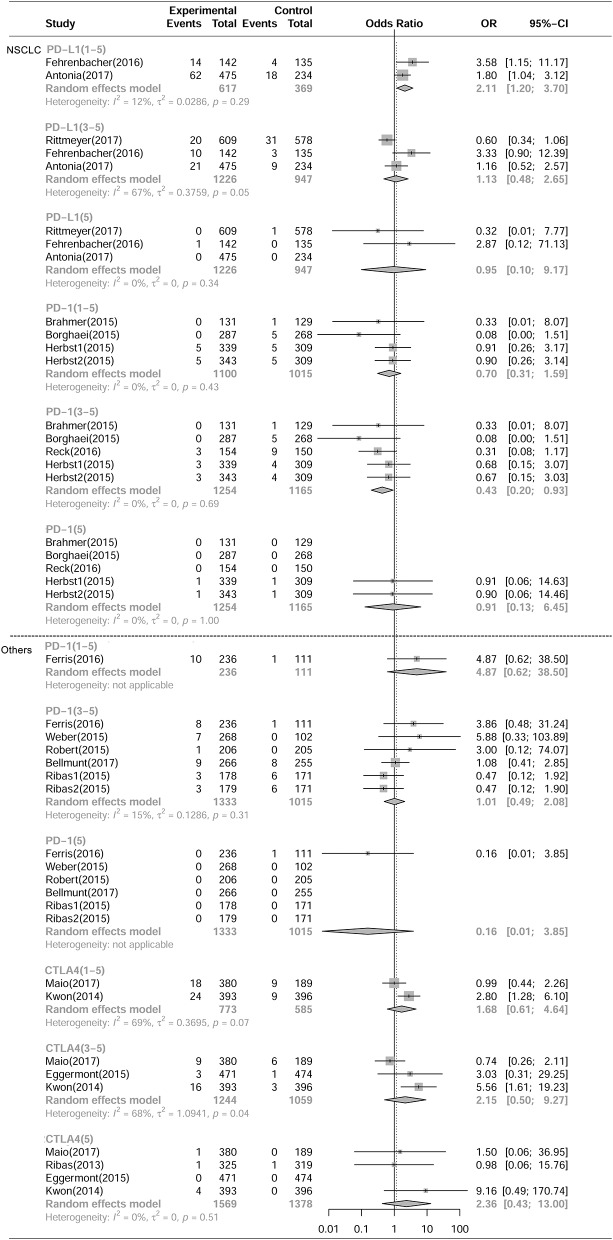
Forest plot analysis of pneumonia of different ICIs in different tumoral types**. NSCLC**, non-small cell lung cancer; **Others**, including MM, HNSCC, Prostate cancer, Mesothelioma PD-L1, PD-L1 inhibitor V.S. chemotherapy/placebo; PD-1, PD-1 inhibitor V.S. chemotherapy; CTLA4, CTLA4 inhibitor V.S. chemotherapy/placebo; **G1-5**, grade 1-5; **G3-5**, grade 3-5, **G5**, death.

As shown in Figures 5, 6, and Table 3, compared with chemotherapy, PD-1 inhibitor treated patients showed significant increase in grade 1-5 and grade 3-5 **pneumonitis** (RR, 4.93, 95% CI: 2.35-10.34, *p* < 0.001; RR, 4.19, 95% CI:1.50-11.76, *p* < 0.001, respectively) in NSCLS subgroup. There was no significant increase in the risk of pneumonia in PD-1 inhibitor treated patients. Similarly, in other tumor types including MM, HNSCC, prostate cancer and mesothelioma, PD-1 inhibitors showed significant increase in grade 1-5 and grade 3-5 pneumonitis (RR, 5.69, 95% CI: 2.00-16.24, *p* < 0.001; RR, 4.05, 95% CI:1.04-15.78, *p* < 0.05, respectively) but no increase in the risk of pneumonia. In NSCLS subgroup, compared with control therapeutics, PD-L1 inhibitors showed significant increase in grade 1-5 **pneumonitis** and **pneumonia** (RR, 3.25, 95% CI: 1.61-6.57, *p* < 0.001; RR, 2.11, 95% CI: 1.20-3.70, *p* < 0.001, respectively). There was no significant difference in the risk of any grade **pneumonitis** and **pneumonia** with CTLA4 inhibitors in other tumor types.

**Table 3:** Risk analysis of pneumonitis and pneumonia of different ICIs in different tumoral types.

	**NSCLC RR, 95%CI (%)**				**Other tumoral types RR, 95%CI (%)**			
	**Pneumonitis (G1-5)**	**Pneumonitis (G3-5)**	**Pneumonia (G1-5)**	**Pneumonia (G3-5)**	**Pneumonitis (G1-5)**	**Pneumonitis (G3-5)**	**Pneumonia (G1-5)**	**Pneumonia (G3-5)**
PD-L1 vs. Control	3.25 (1.61–6.57)^*^	2.16 (0.65–7.20)	NA	NA	NA	NA	2.11 (1.20–3.70)^*^	1.13 (0.48–2.65)
PD−1 vs. Chemo	4.93 (2.35–10.34)^*^	4.19 (1.50–11.76)^*^	0.70 (0.31–1.59)	0.43 (0.20–0.93)^*^	5.69 (2.00–16.24)^*^	4.05 (1.04–15.78)^*^	4.87 (0.62–38.50)	1.01 (0.49–2.08)
CTLA4 vs. Control	NA	NA	NA	NA	6.37 (0.80–50.66)	3.32 (0.55–20.09)	1.68 (0.61–4.64)	2.15 (0.50–9.27)

NSCLC, non-small cell lung cancer; Others, including MM, HNSCC, Prostate cancer, and Mesothelioma; Chemo, chemotherapy; NA, not available; RR, risk ratio; 95%CI, 95% confidence interval.

* *p < 0.05*.

### Heterogeneity of All the Subgroups

There was very tiny overall heterogeneity of grade 1-5/3-5 **pneumonitis** and **pneumonia** incidence in all the remaining subgroups (I^2^ = 0 %. There was statistically significant heterogeneity only in the Nivolumab combined with ipilimumab therapy vs. ipilimumab monotherapy subgroup (I^2^ = 74%, *p* = 0.020).

### Analysis of Publication Bias

Egger's test and Begg's test, conducted by STATA 12.0 software, were utilized to evaluate the publication bias between different RCTs. As presented in Supplementary Table 3 and Supplementary Figure 4, all the *P*-values were > 0.05 after both tests. Therefore, there was no significant publication bias in this meta-analysis.

## Discussion

When combating some solid tumors, ICI therapy alters the balance of immune cells in the body, which in turn cause damage to certain organ system, this phenomenon called immune-related adverse events (irAE) such as pneumonitis, colitis, and endocrine disorders (25, 26). In some randomized clinical trials (RCTs), the reported morbidity for immune-related pneumonitis after ICI inhibitor therapy has been about 1.06(0.53-2.11)% for CTLA4 inhibitors, 3.02(95%CI: 2.31-3.93) % for PD-1 inhibitors and 7.09(95%CI: 5.52-7.16)% for PD-1 combined with CTLA4 inhibitors (2–14, 16–24). ICI-induced pneumonitis has been hypothesized as a state of chronic inflammatory changes, similar to collagen vascular disease which was observed in interstitial pneumonitis (27). Its symptoms were nonspecific, usually manifests through cough, dyspnea, tachypnea, and hypoxia (28). Computed tomography (CT) usually displays reticular infiltrates with ground glass opacities and consolidations in patients with PD-1 inhibitors (29). While, pneumonia after ICI was thought as one conventional AE as that after chemotherapy, which was caused by mainly bacterial infections, whose symptoms were fever, coughing up sputum, and shortness of breath. Its morbidity has varied from 5.47(4.74-7.33) % for CTLA4 inhibitors, 2.23 (1.45-3.39)% for PD-1 inhibitors and 12.36 (9.92-15.29)% for PD-L1 inhibitors observed in solid cancer patients (2, 3, 5–24). In fact, ICI-related pneumonia remains a diagnosis of exclusion because it is difficult to distinguish with regular community acquired pneumonia on the basis of symptoms and imaging alone. In order to better understand the respiratory fatal AEs, we conducted the first meta-analysis on the incidence and difference of pneumonitis and pneumonia following ICIs treatment in solid tumors patients.

Our meta-analysis demonstrated that PD-1/ PD-L1 inhibitors including nivolumab, pembrolizumab, and Atezolizumab could increase the risk of all-grade pneumonitis, which was not seen in CTLA4 inhibitor (ipilimumab). Only pembrolizumab showed significant increase in grade 3-5 pneumonitis compared to chemotherapy. This result is consistent with other reports in that pneumonitis appears to be more common with PD-1/PD-L1 inhibitors (30, 31). Several Clinical trials found no pulmonary toxicities were attributed to the use of CTLA-4 inhibitors (15, 19, 32). The precise pathophysiology of the differences between PD1/PDL-1 inhibitors and CTL-4 inhibitors is yet to be uncovered. Pneumonitis involves dysregulated effector and regulatory T cells in the pulmonary interstitium, ultimately leading to an inflammatory response (33). Some study has shown that PD-1 inhibitors have more potential to activate T cells toward a large spectrum of tissue-specific antigens including lung parenchyma than CTLA4 inhibitors (34, 35). PD-L1 inhibitors seem to have less severe immune-induced toxicicity than PD1 inhibitors partly because PD-L1 blockages do not prevent interactions between PD-L2 and PD-1. PD-L2 might mediate pneumonitis by increasing the interaction with RGMb (repulsive guidance molecule b) expressed by lung interstitial macrophages and alveolar cells, which result in a local T-cell clonal expansion (36). Both nivolumab and pembrolizumab belong to the PD1 inhibitors, but they have different binding areas with PD-1 molecule. The differences in PD-1 binding sites may be associated with the different degree of immune-mediated pneumonitis between pembrolizumab and nivolumab.

Our meta-analysis showed that when combining ipilimumab (CTLA-4 inhibitors) with nivolumab (PD-1 inhibitors), the risk of all-grade pneumonitis (3.47 times), and severe pneumonitis (3.48 times) were higher than nivolumab or ipilimumab alone. Thus, the combined inhibitors of CTLA-4 and PD-1 could generate higher incidences of pneumonitis than either blockade (21–23). It was well known that ICI can activate T cells against tumor cells, and the activated T cells can also attack normal tissues (37) and result in immune-related toxicity of lung. In the meanwhile, the activated immune system may lead to the production of autoantibodies and release of excessive inflammatory cytokines (such as interleukin-17) (38). CTLA-4 inhibitor can attenuate T-cell activation at the early stage in the immune response. On the other hand, PD-1 blockage is able to inhibit T cells at later immune response stage in peripheral tissues. Therefore, we hypothesize that the combined inhibitors of CTLA-4 and PD-1 may synergize lung toxicity than either blockade alone. Further study is needed to uncover the exact molecular mechanism behind this clinical observation.

In this study, we found that PD-L1 inhibitor increased the risk of pneumonia compared to chemotherapy/placebo. However, we did not observed any significant difference in the risk of pneumonia for PD-1 and CTLA4 inhibitors monotherapy (vs. chemotherapy), and combination therapy (vs. monotherapy). Interestingly, patients with PD-L1 inhibitors treatment for NSCLC had a higher incidence of pneumonia than those patients with other cancer types. In one American analysis of data from 11,111 lung cancer patients and 49,975 patients with other solid tumors, pneumonia occurred more commonly in the lung cancer patients (26.4 vs. 10.3%) (39). Therefore, pre-existing damage to the lung tissue by the tumor might predispose the lung to treatment side effects.

In reality, both pneumonitis and pneumonia were uncommon. Potentially serious lung toxicities occur in only 1–7 and 1–5% of ICI-treated patients, respectively. Most cases of pneumonitis and pneumonia were mild. With the increasing use of ICIs in many anticancer settings, the absolute burden and mortality of pneumonitis and pneumonia will undoubtedly rise. This meta-analysis study provided new information to physicians regarding the difference of pneumonitis and pneumonia in cancer patients treated with ICIs, especially those with PD-1 and CTLA4 inhibitors. Further study on the molecular mechanisms underlying these side effects of ICI therapy could help us to implement a better therapeutic strategy and to avoid some of these side effects (40).

This type of meta-analysis itself based on published data had several unavoible limitations. First, this meta-analysis did not include individual patient data, the use of which would have provided more details about pneumonitis and pneumonia with ICIs. Secondly, the sensitivity analysis was not employed in this meta-analysis because of the paucity of the study number on pneumonia with nivolumab and ipilimumab vs. ipilimumab. Thirdly, the clinical studies included were not specifically designed to assess the immune related pneumonitis and pneumonia, and a generally acknowledge of the diagnostic criteria is still lacking. Cancer patients are generally prune to have lung infection due to overall health condition and the compromised immune system. Some lung infections or tumor progression could have been misdiagnosed clinically as pneumonitis. Therefore, the identification of autoimmune pneumonitis in these studies may not be completely accurate or homogeneous. Moreover, the final diagnosis of immune pneumonitis was not central reviewed, so it could depend on the experience of every center. Prospective centrally reviewed multicenter studies with more stringent diagnostic criteria could help us better understand the relative risk and the pathogenesis of these immune related pulmonary complications. On the other hand, we have made great efforts on the overall quality assessment to make our conclusion more steady and credible: (1) two independent reviewers searched all the relevant trails with well-defined inclusion criteria. They assessed studies appropriate for meta-analysis evaluated by using PICO chart and assessed the risk of bias for the included RCTs according to the Cochrane Handbook. (2) Two independent reviewers verified data in our meta-analysis which was performed by pair-wise comparisons. (3) The random-effects model and subgroup analysis were employed statistically in this meta-analysis. (4) The heterogeneity of nearly all the subgroups was low or moderate.

## Conclusion

In summary, our meta-analysis has demonstrated that PD-1/ PD-L1 inhibitors including nivolumab, pembrolizumab and atezolizumab could increase the risk of all-grade pneumonitis rather than CTLA4 inhibitor (ipilimumab). Only pembrolizumab showed the significant increase in grade 3-5 pneumonitis compared to chemotherapy. The risk of Grade 1-5/3-5 pneumonitis of combined ipilimumab with nivolumab was higher than nivolumab/ipilimumab alone. PD-L1 inhibitor may increase the risk of pneumonia compared to chemotherapy/placebo. Clinicians need to be aware of these ICI-associated respiratory disorders when employing ICI therapy for solid tumors so that patients can be appropriately managed.

## Methods

The systematic review with meta-analysis was conducted according to the guidelines of the Cochrane Handbook for Systematic Reviews of Interventions (41), and reported according to the PRISMA Statement (40) (Supplementary Table 1).

### Searching Strategy

We searched the following databases: PubMed, Embase and https:// clinicaltrials.gov. (up to January 7, 2018) for studies reporting the risk of pneumonitis or pneumonia associated with ICIs monotherapy vs. chemotherapy or control, combination therapy (PD-1 inhibitor plus CTLA4 inhibitor) vs. ICI single therapy, and combination therapy (ICIs plus chemotherapy) vs. chemotherapy for the treatment of patients with solid tumors. The medical subject heading (MeSH) terms included in searching the relevant studies contained one term that means neoplasms (neoplasm, carcinoma, cancer, or tumor, etc), one term means ICIs (anti-CTLA-4, anti-PD-1, anti-PD-L1, ipilimumab, tremelimumab, pembrolizumab, nivolumab, durvalumab, atezolizumab, or avelumab etc), and one term related to randomized controlled trials (RCTs). We used “and” to connect the terms (Supplementary Table 2).

### Inclusion Criteria

Studies in English literature with the following information were included in our meta-analysis: (1) Phase II/III RCTs with primary endpoints including overall survival (OS), progression-free survival (PFS), or objective response rate (ORR); (2) histologically confirmed solid cancer such as lung cancer, and others; (3) containing the information of ICIs (PD-1/PD-L1 inhibitor or CTLA4 inhibitor alone or PD-1 inhibitor combined with CTLA4 inhibitor), controlled therapies, pneumonitis, and pneumonia.

The studies were excluded if they were: (1) letters, reviews, unfinished studies, duplicate reports, or conference reports; (2) studies conducted with animal models or cell lines; (3) studies due to insufficient data; (4) papers in other languages than English; (5) RCTs in phase I.

## Data Extraction

Two independent reviewers (**Y.L.H. and Q.S**.) searched all the relevant studies and read the titles, abstracts, and full texts of the identified studies. We accessed each study appropriateness for meta-analysis by using the PICO (patient, intervention, comparison and outcome) chart (42). The following information was extracted from the selected studies: year of publication, name of journal, the last name of the first author, treatment arms, the primary endpoint, type of underlying solid tumor, number of patients in the ICIs treatment groups, number of patients in control groups, number of patients bearing pneumonitis or pneumonia of all-grade (grade 1-5), high-grade (grade 3-5), and death (grade 5; Table 2). Disagreements in assessing the cases or data were resolved via discussion with the third reviewer **(X.C.Z.)**.

### Data Analysis

In the meta-analysis, the risk of bias analysis of all included studies was performed using Review Manager 5.3 software (Cochrane Collaboration 2014, Nordic Cochrane Center, Copenhagen, Denmark). Two reviewers (**Q.S. and Y.L.H**.) independently assessed the quality of the included RCTs according to the Cochrane risk of bias tool, which assesses the following seven domains: selection bias (including both random sequence generation and allocation concealment), performance bias, detection bias, attrition bias, reporting bias, and other bias. R3.4.3 (R Project). The metafor package software was used for our meta-analysis. The Risk Ratio (RR) was used to estimate overall and severe pneumonitis or pneumonia (grade 1-5/3-5/5). RR >1.0 indicates higher risk or higher incidence of overall and severe pneumonitis or pneumonia in patients treated with ICIs than those treated with chemotherapy or placebo. In addition, the Q test and I^2^ statistics were used to assess the heterogeneity among the RCTs. I^2^ values of <30, 30–59, 60–75, and >75% were classified as low, moderate, substantial, and considerable heterogeneity, respectively. (43) We used the random-effects model described by DerSimonian and Laird (44) to calculate pooled RR and 95% confidence interval (CI). Sensitivity analysis was performed by removing one study at a time, to examine whether the results could have been influenced by a single study, especially in those studies with dubious results or considerable heterogeneity. Sources of heterogeneity were explored using subgroup analyses according to different ICIs or cancer types. The Begg's and Egger's tests were used to analyze the publication bias across RCTs. All *P*-values were 2-tailed, and a probability level <0.05 was considered statistically significant.

### Quality Assessment

PICO chart was used to assess study's appropriateness for meta-analysis. Cochrane Handbook for Systematic Reviews of Interventions was used to assess the risk of bias for the included studies. All disagreements in our meta-analysis were resolved by discussion with the third reviewer (**X.C.Z**.). Subgroup analysis and sensitivity analysis were used to assess the heterogeneity among the RCTs. Random-effects model (REM) was employed to validate the statistical results in our meta-analysis.

## Author Contributions

QS and EZ had access to all the data included in the study and are responsible for the completeness of the data and the accuracy of our analysis. JW, YH and TL helped to design the study. QS, DW, and YH contributed to the statistical analysis and the revision of this manuscript. QS and ZG approved the final manuscript.

### Conflict of Interest Statement

The authors declare that the research was conducted in the absence of any commercial or financial relationships that could be construed as a potential conflict of interest.
