# Supplementary material for: Risk of Pneumonitis and Pneumonia Associated With Immune Checkpoint Inhibitors for Solid Tumors: A Systematic Review and Meta-Analysis
Source: Front Immunol. 2019 Feb 4;10:108. doi: 10.3389/fimmu.2019.00108 (PMC6369169; doi:10.3389/fimmu.2019.00108)
Supplement: Supplementary file 1 [file Data_Sheet_1.docx]

**Supplementary Table 1: Checklist of items included when reporting a systematic review and meta- analysis**

| **Section/topic** | **Item No** | **Checklist item** | **Reported on page No** |
| --- | --- | --- | --- |
| **Title** |  |  |  |
| Title | 1 | Identify the report as a systematic review, meta-analysis, or both | 1 |
| **Abstract** |  |  |  |
| Structured summary | 2 | Provide a structured summary including, as applicable, background, objectives,data sources, study eligibility criteria, participants, interventions, study appraisaland synthesis methods, results, limitations, conclusions and implications of key findings, systematic review registration number | 3 |
| **Introduction** |  |  |  |
| Rationale | 3 | Describe the rationale for the review in the context of what is already known | 4-5 |
| Objectives | 4 | Provide an explicit statement of questions being addressed with reference to participants, interventions, comparisons, outcomes, and study design (PICOS) | 5 |
| **Methods** |  |  |  |
| Protocol and registration | 5 | Indicate if a review protocol exists, if and where it can be accessed (such as web address), and, if available, provide registration information including registration number |  |
| Eligibility criteria | 6 | Specify study characteristics (such as PICOS, length of follow-up) and report characteristics (such as years considered, language, publication status) used as criteria for eligibility, giving rationale | 6 |
| Information sources | 7 | Describe all information sources (such as databases with dates of coverage, contact with study authors to identify additional studies) in the search and date last searched | 6-7 |
| Search | 8 | Present full electronic search strategy for at least one database, including any limits used, such that it could be repeated | 7 |
| Study selection | 9 | State the process for selecting studies (that is, screening, eligibility, included in systematic review, and, if applicable, included in the meta-analysis) | 7 |
| Data collection process | 10 | Describe method of data extraction from reports (such as piloted forms, independently, in duplicate) and any processes for obtaining and confirming data from investigators | 7 |
| Data items | 11 | List and define all variables for which data were sought (such as PICOS, funding sources) and any assumptions and simplifications made | 8 |
| Risk of bias in individual studies | 12 | Describe methods used for assessing risk of bias of individual studies (including specification of whether this was done at the study or outcome level), and how this information is to be used in any data synthesis | 8 |
| Summary measures | 13 | State the principal summary measures (such as risk ratio, difference in means). | 8 |
| Synthesis of results | 14 | Describe the methods of handling data and combining results of studies, if done, including measures of consistency (such as I2 statistic) for each meta analysis | 8 |
| Risk of bias across studies | 15 | Specify any assessment of risk of bias that may affect the cumulative evidence (such as publication bias, selective reporting within studies) | 8 |
| Additional analyses | 16 | Describe methods of additional analyses (such as sensitivity or subgroup analyses, meta-regression), if done, indicating which were pre-specified | 8 |
| **Results** |  |  |  |
| Study selection | 17 | Give numbers of studies screened, assessed for eligibility, and included in the review, with reasons for exclusions at each stage, ideally with a flow diagram | 8-9 |
| Study characteristics | 18 | For each study, present characteristics for which data were extracted (such as study size, PICOS, follow-up period) and provide the citations | 9 |
| Risk of bias within studies | 19 | Present data on risk of bias of each study and, if available, any outcome-level assessment (see item 12). | 9-10 |
| Results of individual studies | 20 | For all outcomes considered (benefits or harms), present for each study (a) simple summary data for each intervention group and (b) effect estimates and confidence intervals, ideally with a forest plot | 9-11 |
| Synthesis of results | 21 | Present results of each meta-analysis done, including confidence intervals and measures of consistency | 9-11 |
| Risk of bias across studies | 22 | Present results of any assessment of risk of bias across studies (see item 15) | 11 |
| Additional analysis | 23 | Give results of additional analyses, if done (such as sensitivity or subgroup analyses, meta-regression) (see item 16) |  |
| **Discussion** |  |  |  |
| Summary of evidence | 24 | Summarise the main findings including the strength of evidence for each main outcome; consider their relevance to key groups (such as health care providers, users, and policy makers) | 12-15 |
| Limitations | 25 | Discuss limitations at study and outcome level (such as risk of bias), and at review level (such as incomplete retrieval of identified research, reporting bias) | 15 |
| Conclusions | 26 | Provide a general interpretation of the results in the context of other evidence, and implications for future research | 16 |
| **Funding** |  |  |  |
| Funding | 27 | Describe sources of funding for the systematic review and other support (such as supply of data) and role of funders for the systematic review | 16 |

**Supplementary Table 2: Searches performed in Pubmed and Embase**

| ***PubMed*** | | | |
| --- | --- | --- | --- |
| ***#1*** | (((((((((Neoplasms) OR Neoplasia) OR Neoplasias) OR Neoplasm) OR Tumors) OR Tumor) OR Malignancy) OR Malignancies) OR Cancer) OR Cancers | | ***4029458*** |
| ***#2*** | (((((((((nivolumab) OR MDX-1106) OR ONO-4538) OR BMS-936558) OR Opdivo))  OR  ((((pembrolizumab) OR lambrolizumab) OR Keytruda) OR MK-3475)))  OR  (((Durvalumab)  OR  (((((((atezolizumab) OR anti-PDL1) OR immunoglobulin g1,anti (human cd antigens cd274) (human monoclonal mdpl3280a heavy chain) , disulfide with human monoclonal mdpl3280a kappa-chain, dimer) OR MPDL3280A) OR tecentriq) OR RG7446) OR RG-7446))  OR  ((avelumab) OR MSB0010718C)))  OR  (((((((((((tremelimumab) OR ticilimumab) OR CP 675) OR CP675 cpd) OR CP-675) OR CP-675,206) OR CP-675206) OR CP675206) OR CP 675206))  OR  ((((((((((Ipilimumab) OR Anti CTLA 4 MAb Ipilimumab) OR Anti-CTLA-4 MAb Ipilimumab) OR Ipilimumab, Anti-CTLA-4 MAb) OR Yervoy) OR MDX010) OR MDX 010) OR MDX-010 M) OR DX-CTLA-4) OR MDX CTLA 4 | | ***8493*** |
| ***#3*** | (((((((randomized controlled trail) OR controlled clinicaltrail) OR randomized) OR randomly) OR trial)) | | ***949148*** |
| ***#4*** | *#1 AND #2 AND #3* | | ***Final search***  ***1267*** |
| ***EMBASE*** | | | |
| ***#1*** | acral AND ('tumor'/exp OR tumor) OR 'neoplasms'/exp OR neoplasms OR (acral AND ('tumour'/exp OR tumour)) OR (('neoplasms'/exp OR neoplasms) AND by AND histologic AND type) OR (neoplasms, AND cystic, AND mucinous, AND serous) OR (neoplasms, AND embryonal AND mixed) OR (neoplasms, AND ('germ'/exp OR germ) AND ('cell'/exp OR cell) AND embryonal;) OR (neoplasms, AND glandular AND epithelial;) OR (neoplasms, AND 'hormone dependent;') OR (neoplasms, AND 'post traumatic') OR (neoplastic AND ('disease'/exp OR disease)) OR 'tumor'/exp OR tumor OR 'tumour'/exp OR tumour | ***4766257*** | |
| ***#2*** | 'ipilimumab'/exp OR ipilimumab OR (bms AND 734016;) OR bms734016; OR (mdx AND 010) OR (mdx AND 101;) OR 'mdx010'/exp OR mdx010 OR 'mdx101'/exp OR mdx101 OR strentarga; OR 'yervoy'/exp OR yervoy  OR  'ticilimumab'/exp OR ticilimumab OR (cp AND 675, AND 206) OR (cp AND 675206) OR (cp AND 675 AND 206) OR (cp675, AND 206) OR 'cp675206'/exp OR cp675206 OR 'tremelimumab'/exp OR tremelimumab  OR  'nivolumab'/exp OR nivolumab OR (bms AND 936558) OR 'bms936558'/exp OR bms936558 OR (mdx AND 1106) OR (ono AND 4538) OR 'mdx1106'/exp OR mdx1106 OR 'ono4538'/exp OR ono4538 OR 'opdivo'/exp OR opdivo  OR  'pembrolizumab'/exp OR pembrolizumab OR 'keytruda'/exp OR keytruda OR 'lambrolizumab'/exp OR lambrolizumab OR (mk AND 3475) OR 'mk3475'/exp OR mk3475 OR  'avelumab'/exp OR avelumab OR bavenci OR (msb AND 0010718) OR (msb AND 10682) OR (msb AND 0010682) OR msb0010718c; OR msb10682; OR 'msb10718c'/exp OR msb10718c OR  'atezolizumab'/exp OR atezolizumab OR (monoclonal AND ('antibody'/exp OR antibody) AND mpdl AND 3280a) OR (monoclonal AND ('antibody'/exp OR antibody) AND ('mpdl3280a'/exp OR mpdl3280a)) OR (mpdl AND 3280a) OR 'mpdl3280a'/exp OR mpdl3280a OR (rg AND 7446) OR 'rg7446'/exp OR rg7446 OR 'tecentriq'/exp OR tecentriq OR 'tecntriq'/exp OR tecntriq OR  'durvalumab'/exp OR durvalumab OR (medi AND 4736) OR 'medi4736'/exp OR medi4736 | ***14326*** | |
| ***#3*** | randomized AND controlled AND ('trial'/exp OR trial) OR (controlled AND trial, AND randomized;) OR 'randomized controlled trial'/exp OR 'randomized controlled trial' OR (pragmatic AND ('clinical'/exp OR clinical) AND trials) OR (randomised AND controlled AND ('study'/exp OR study)) OR (randomised AND controlled AND ('trial'/exp OR trial)) OR (randomized AND controlled AND study;) OR (trial, AND randomized AND controlled) | ***755544*** | |
| ***#4*** | ***#1 AND #2 AND #3*** | ***Final search***  ***2169*** | |

**Supplementary Figure 1: Flowchart depicting the RCTs selection process.**

**
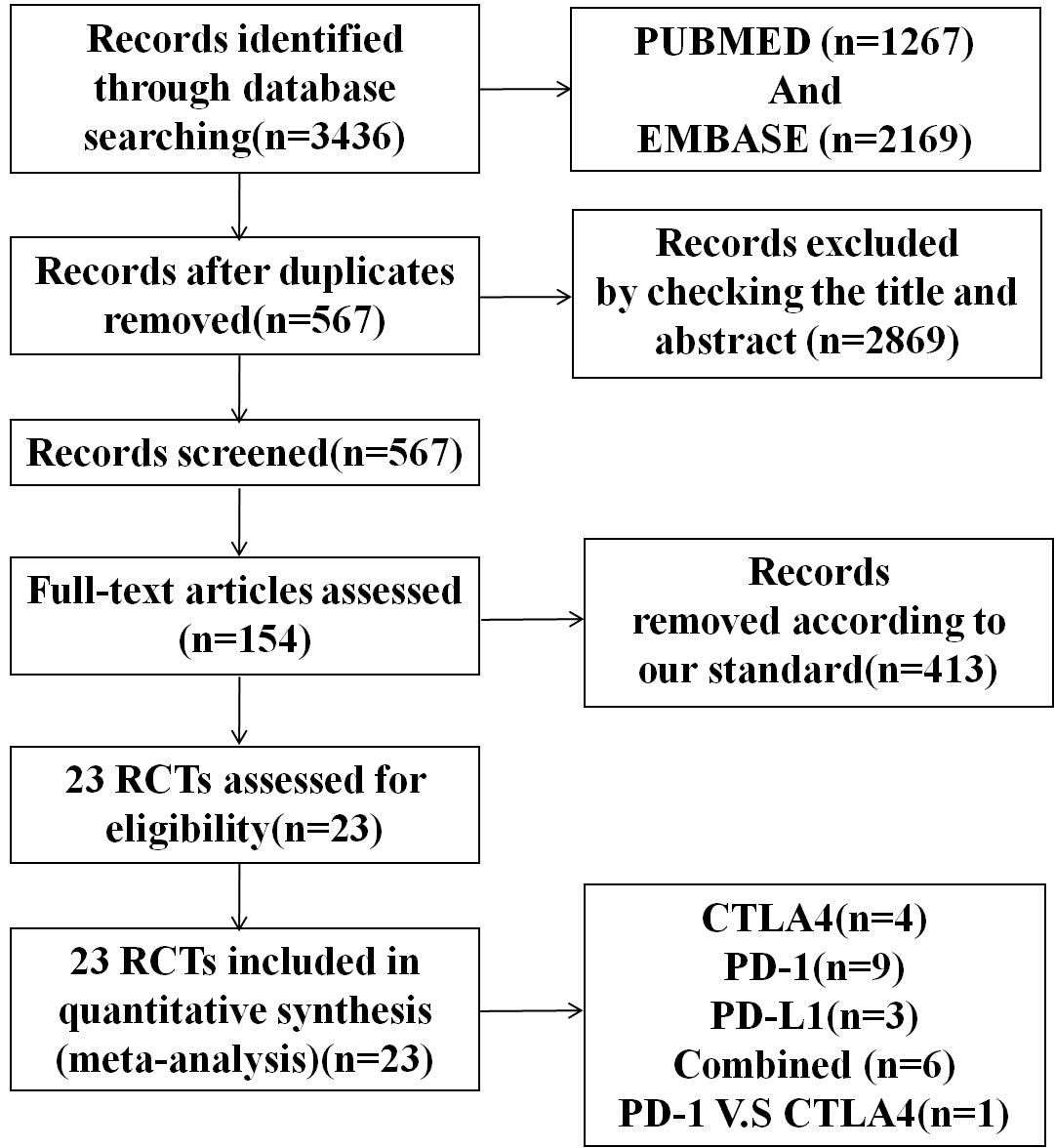
**

**Supplementary Figure 2: Risk of bias summary**

**
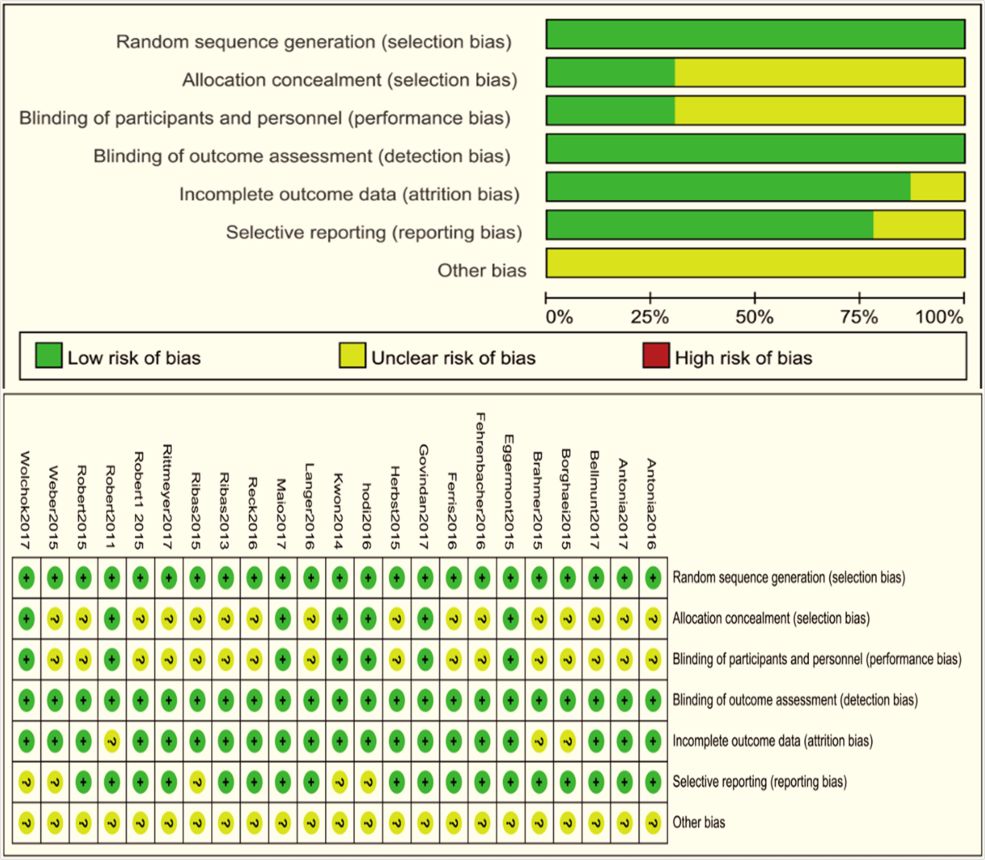
**

**Notes:** (**A**): Bar chart comparing the percentage risk of bias for each included RCT. Low risk of bias (Green), high risk of bias (Red), and unclear risk of bias (Yellow). (**B**): Risk of bias for each included RCT, representing low risk of bias (+), high risk of bias (-), and unclear risk of bias (?).

**Supplementary Figure 3: Forest plot analysis of pneumonitis and pneumonia comparing different ICIs with control therapies
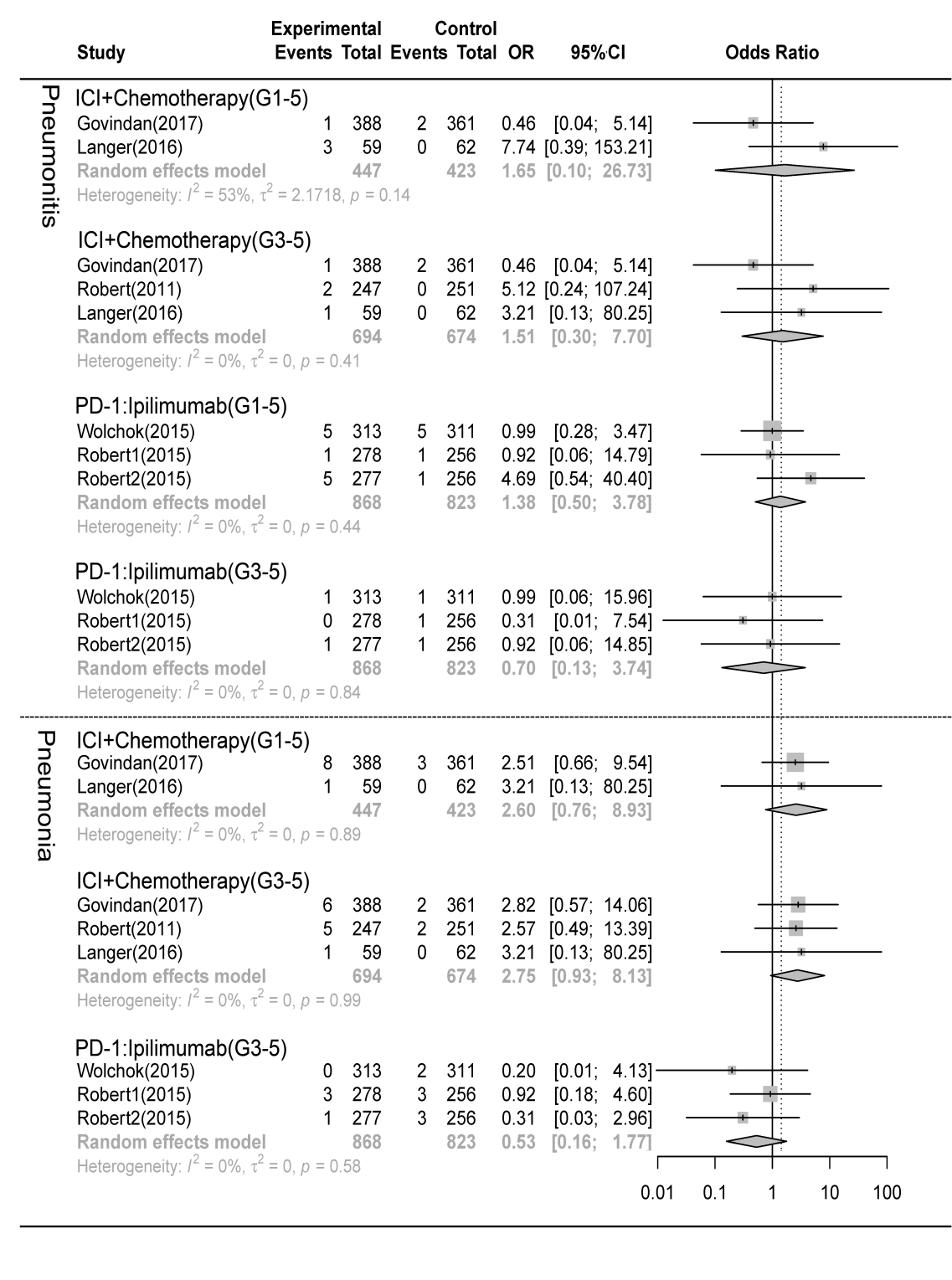
**

**Notes: ICI+Chemotherapy**: ICI+chemotherapy V.S. chemotherapy; **PD-1: Ipilimumab**: PD-1 inhibitor V.S. ipilimumab; **G1-5**: grade1-5; **G3-5**: grade3-5

**Supplementary Table 3: Publication bias in the PD1 group with Begg’s and Egger's tests**

|  | Trials | Heterogeneity | | RR（95%CI） | Begg’s test | | Egger’s test | |
| --- | --- | --- | --- | --- | --- | --- | --- | --- |
|  |  | P | I^2^ |  | Z | P | t | P |
| Pneumonitis(G1-5) | 9 | 0.997 | 0.0% | 0.88(0.34,2.27) | 0.08 | 0.94 | 1.32 | 0.22 |
| Pneumonitis(G3-5) | 9 | 0.996 | 0.0% | 0.71(0.43,1.16) | -1.52 | 1.43 | -1.17 | 0.87 |
| Pneumonia(G1-5) | 9 | 0.243 | 26.8% | 5.02(2.76,9.13) | 0.31 | 0.76 | -0.24 | 1.00 |
| Pneumonia (G3-5) | 9 | 0.399 | 4.6% | 4.09(1.81,9.26) | 0.89 | 0.37 | 0.26 | 0.81 |

**G1-5:** grade1-5; **G3-5:** grade3-5.

**Supplementary figure 4: Begg's funnel plot**

**1.1 Begg's funnel plot for pneumonitis (Grade1-5) in patients treated with *PD-1 inhibitors***


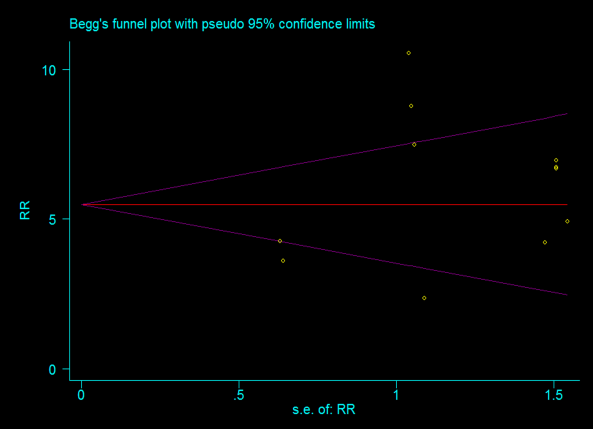


**1.2 Begg's funnel plot for pneumonitis (Grade3-5) in patients treated with *PD-1 inhibitors***


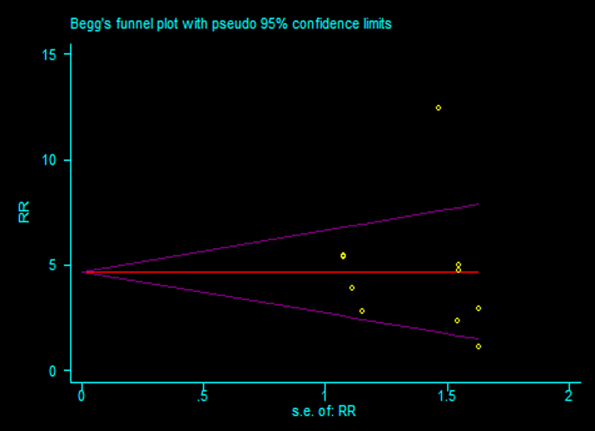


**2.1 Begg's funnel plot for pnuemonia (Grade1-5) in patients treated with *PD-1 inhibitors***


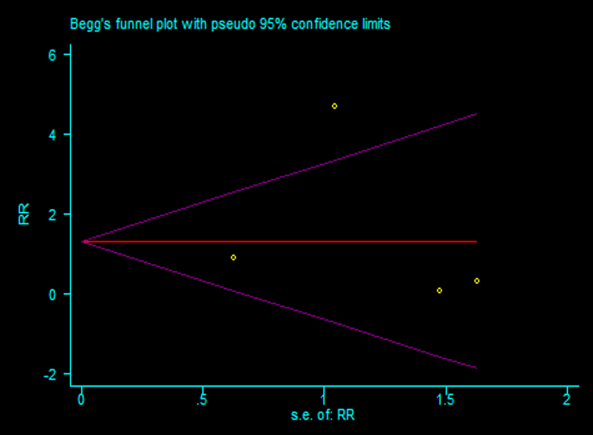


**2.2 Begg's funnel plot for pnuemonia (Grade3-5) in patients treated with *PD-1 inhibitors***
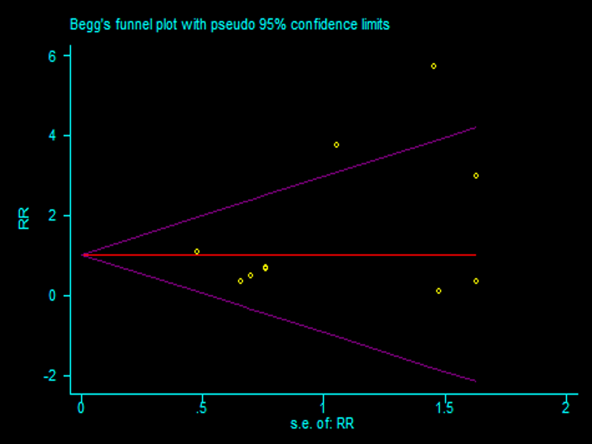


**Abbreviation list:**

**PD-1:** programmed cell death protein 1

**PD-L1:** programmed cell death protein ligand 1

**PD-L2:** programmed cell death protein ligand 2

**CTLA4:** cytotoxic T-lymphocyte-associated protein 4

**ICI:** immune checkpoint inhibitor

**FDA:** Food and Drug Administration

**irAE:** immune-related adverse events

**RCT:** randomized controlled trials

**PFS:** progression-free survival

**OS:** overall survival;

**ORR:** objective response rate;

**MM:** melanoma;

**NSCLC:** non-small cell lung cancer;

**SCLC:** small cell lung cancer;

**HNSCC:** head-neck squamous cell carcinoma

**PICO:** patient, intervention, comparison and outcome

**Nivo:** nivolumab

**Ipi:** ipilimumab

**Pem:** pembrolizumab

**Versus:** VS

**Chem:** cheotherapy

**95%CI:** 95% confidence interval

**RR:** Risk Ratio

**FEM:** fixed-effects model

**REM:** random-effects model

**DTIC:** Dacarbazin

**DOX:** docetaxel

**NA:** Not available.
